# Targeting RANKL Prevents Bone Loss, Improves Muscle Function and Extends Lifespan in Progeroid Mice

**DOI:** 10.1111/acel.70655

**Published:** 2026-08-09

**Authors:** Sandra Freitas‐Rodríguez, Alejandra Valle‐Cao, Francisco Rodríguez, Manuel Fernández‐Sanjurjo, Benjamín Fernández‐García, Vanessa Loredo, María Teresa Fernández‐García, Carmen Fiuza‐Luces, Alejandro Lucia, Carlos López‐Otín, Alejandro López‐Soto, Alicia R. Folgueras

**Affiliations:** ^1^ Departamento de Bioquímica y Biología Molecular, Facultad de Medicina, Instituto Universitario de Oncología del Principado de Asturias (IUOPA) Universidad de Oviedo Oviedo Spain; ^2^ Unidad de Transgénicos, Servicios Científico‐Técnicos. Bioterio Universidad de Oviedo Oviedo Spain; ^3^ Departamento de Biología Funcional, Área de Fisiología, Facultad de Medicina Universidad de Oviedo Oviedo Spain; ^4^ Instituto de Investigación Sanitaria del Principado de Asturias (ISPA) Oviedo Spain; ^5^ Departamento de Morfología y Biología Celular, Facultad de Medicina Universidad de Oviedo Oviedo Spain; ^6^ Departamento de Medicina, Área de Pediatría, Facultad de Medicina Universidad de Oviedo Oviedo Spain; ^7^ Unidad de Histopatología Molecular, IUOPA Universidad de Oviedo Oviedo Spain; ^8^ Physical Activity and Health Research Group Research Institute of the Hospital 12 de Octubre Madrid Spain; ^9^ Department of Sport Sciences, Faculty of Medicine, Health and Sports Universidad Europea de Madrid Madrid Spain; ^10^ CIBER of Frailty and Healthy Aging Carlos III Health Institute Madrid Spain; ^11^ Facultad de Ciencias de la Vida y la Naturaleza Universidad Nebrija Madrid Spain

**Keywords:** aging, bone loss, denosumab, HGPS, progeria, RANKL

## Abstract

Hutchinson‐Gilford progeria syndrome (HGPS) is a rare genetic disorder characterized by the early development of pathological features associated with aging, ultimately leading to premature death. HGPS primarily affects tissues of mesenchymal origin, as evidenced by the clinical manifestations characteristic of this premature aging disorder, including, but not limited to, osteoporosis, muscle wasting, lipodystrophy, and cardiovascular disease. In this study, we used preclinical mouse models and both genetic and translational approaches to investigate whether an antiresorptive strategy, based on RANKL targeting, ameliorated the bone loss phenotype of progeroid mice. Here we show that osteocyte‐derived RANKL deletion in the *Zmpste24* ^*−/−*^ mouse model of HGPS reverted bone loss in both long bones and vertebrae. These mice also exhibited increased grip strength and improved endurance capacity. Furthermore, *Zmpste24* ^*−/−*^ mice showed increased survival upon osteocyte‐specific RANKL deletion. Notably, the use of a translational approach based on the administration of a neutralizing antibody against RANKL also restored bone mass, reduced muscle fibrosis, and extended the lifespan of *Zmpste24* ^*−/−*^ mice. Altogether, these findings support that targeting RANKL exerts a beneficial effect on both osseous and extra‐osseous phenotypes of HGPS, suggesting the potential of this therapeutic approach to explore in the treatment of this disease.

## Graphical abstract

Targeting of RANKL by genetic and pharmacological approaches ameliorates key features of the progeroid phenotype in *Zmpste24* ^*−/−*^ mice. RANKL intervention restores bone mass, improves muscle phenotype, and extends survival. These findings support further exploration of RANKL‐targeted therapies for Hutchinson‐Gilford progeria syndrome. Graphical Abstract was created with BioRender.com.

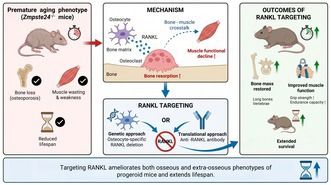

## 1 Introduction

Loss of bone mass and muscle weakness are among the many clinical manifestations associated with the aging process. The structure and function of the skeleton depend on the active renewal of bone tissue, which involves a finely tuned equilibrium between the resorption of old bone by osteoclasts and the deposition of newly formed tissue by osteoblasts, in a process known as bone remodeling (Bolamperti et al. 2022). Several factors related to physiological aging disrupt the local and systemic bone microenvironment and have been associated with the imbalance in bone homeostasis, leading to the development of osteoporosis in older people. The structural association between bone and muscle establishes a tight crosstalk that determines the correct function of both tissues. Several studies have shown a significant association between sarcopenia (i.e., the decline in muscle mass and function) and osteoporosis in the context of aging (Kirk et al. 2020), supporting increasing evidence of shared mechanical, molecular and cellular mechanisms in the regulation of bone and skeletal muscle function under physiological and pathological conditions (Bonewald 2019).

Denosumab, a neutralizing antibody against the cytokine TNFSF11—best known as RANKL (receptor activator of nuclear factor kappa‐Β ligand)—is one of the anti‐resorptive approaches currently being used for the treatment of osteoporosis (Kendler et al. 2022). The binding of RANKL to its membrane receptor RANK is essential for osteoclast function and bone resorption. However, recent studies have demonstrated that RANKL also exerts relevant roles beyond bone metabolism regulation. Both RANK and RANKL are expressed in skeletal muscle tissue, and denosumab treatment has been shown to improve muscle function in addition to bone mass in preclinical models that develop muscular dystrophy or sarcopenia, highlighting the potential relevance of therapeutic strategies targeting RANKL for the treatment of musculoskeletal pathologies involving both loss of bone mass and muscle dysfunction (Bonnet et al. 2019; Dufresne et al. 2018; Jayash et al. 2023).

Hutchinson‐Gilford progeria syndrome (HGPS) is a rare genetic disorder characterized by the development of a premature aging phenotype during childhood. Early death of patients with HGPS, with an average lifespan of 14.6 years, is mainly attributed to severe pathological changes observed in their cardiovascular system, including atherosclerosis, calcium deposition, and vascular stiffness, ultimately leading to myocardial infarction or stroke (Hennekam 2006). The clinical manifestations that define this syndrome also recapitulate other symptoms associated with natural aging, including alopecia, reduced bone density, joint contractures, lipodystrophy, and muscle wasting, in addition to growth impairment (Gordon et al. 2014; Hennekam 2006). HGPS is primarily caused by a *de novo* point mutation (c.1824C>T, p.G608G) in the *LMNA* gene, which encodes lamin A and lamin C isoforms. This specific mutation activates a cryptic splice donor site, resulting in the loss of an amino acid sequence that is essential for the post‐translational cleavage and removal of the farnesylated C‐terminal end of prelamin A by the zinc metallopeptidase STE24 (ZMPSTE24) (Eriksson et al. 2003). As a result, this mutation leads to the accumulation of an aberrant farnesylated lamin A isoform called progerin, which causes defects in the nuclear envelope assembly, eventually leading to the multiple pathological phenotypes of HGPS (Gordon et al. 2014). Other progeroid laminopathies, such as Mandibuloacral dysplasia type B (MADB) and Restrictive dermopathy (RD), are caused by mutations in the *ZMPSTE24* gene, which lead to the accumulation of farnesylated prelamin A. The extent to which these mutations impair ZMPSTE24 enzymatic activity determines the severity of the clinical manifestations observed in these human disorders, with the most severe outcome reported in RD, where homozygous *ZMPSTE24* null mutations cause perinatal lethality (Cenni et al. 2018). Despite heterogeneity in disease course, progeroid laminopathies share several features, including severe skeletal phenotypes characterized by generalized osteoporosis, osteolysis of the distal phalanges and clavicles, mandibular hypoplasia, and growth retardation. These laminopathies therefore provide a relevant framework for dissecting the molecular and cellular mechanisms underlying bone disease and for guiding the development of novel intervention strategies targeting skeletal diseases (Gargiuli et al. 2018).

Considering the low prevalence of premature aging disorders, the generation and use of preclinical mouse models has been fundamental in gaining insight into the molecular basis of progeroid syndromes and developing potential therapeutic approaches (Folgueras et al. 2018). Among the several mouse models that have been generated to date, *Zmpste24* ^*−/−*^ mice, which lack the metallopeptidase involved in lamin A maturation, recapitulate many of the phenotypes observed in patients with HGPS and MAD, particularly those related to musculoskeletal alterations. In this study, we used the *Zmpste24* ^*−/−*^ mouse model to explore whether therapeutic strategies based on RANKL inhibition, which have been shown to be effective in the treatment of age‐related osteoporosis, could improve the bone loss phenotype and muscle function in the context of progeroid laminopathies.

## 2 Results

### 2.1 Deletion of Osteocyte RANKL Prevents Osteoporotic Phenotype in *Zmpste24* ^*−/−*^ Mice

**FIGURE 1:**
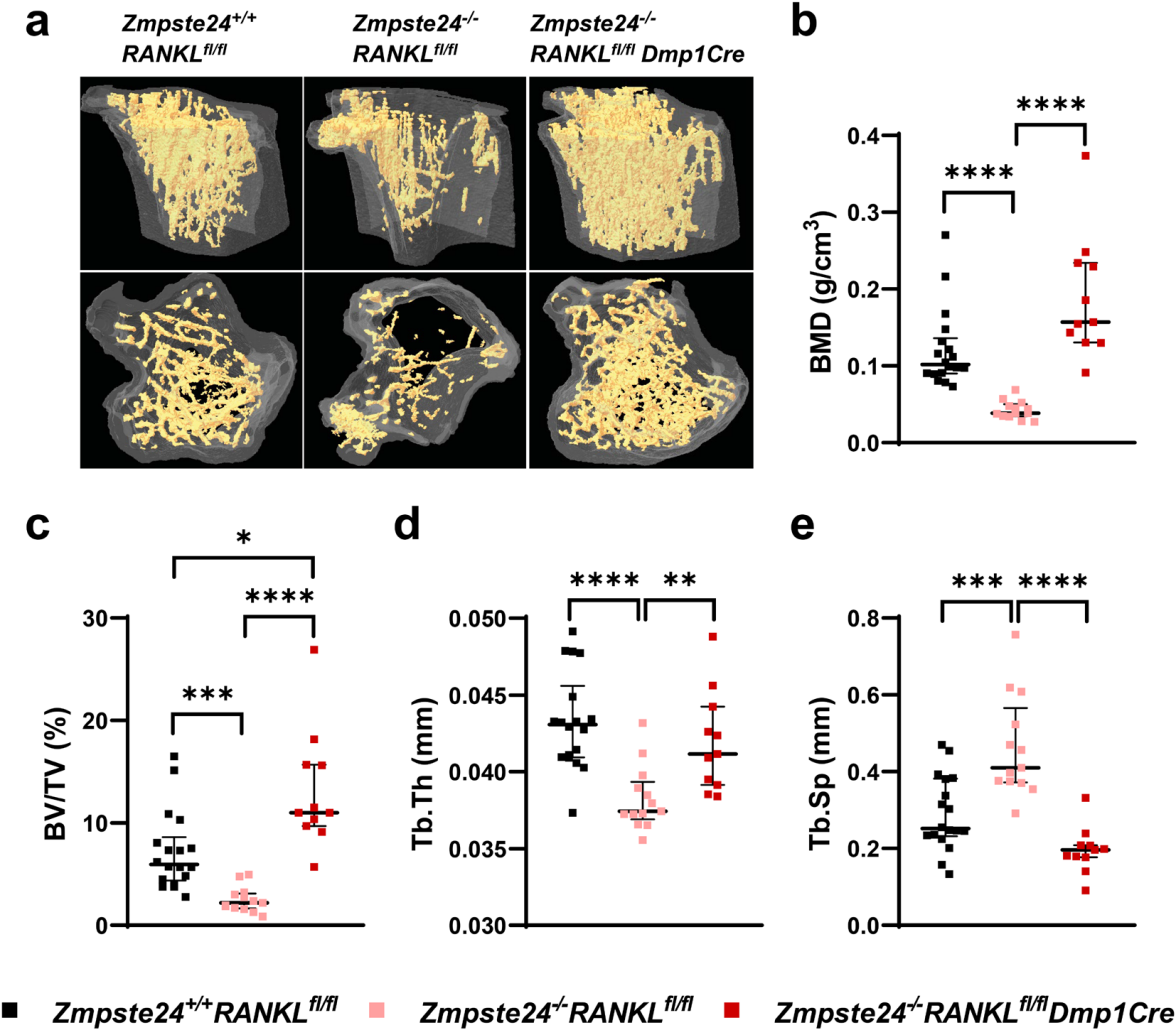
Deletion of osteocyte‐derived RANKL in *Zmpste24*‐deficient mice prevents bone loss. (a) Representative micro‐computed tomography images of the distal tibia from ~6.5–month‐old *Zmpste24* ^*+/+*^ */RANKL* ^*fl/fl*^, *Zmpste24* ^*−/−*^ */RANKL* ^*fl/fl*^ and *Zmpste24* ^*−/−*^ */RANKL* ^*fl/fl*^ */Dmp1‐Cre* littermates. (b) Bone mineral density (BMD); (c) bone volume relative to tissue volume (BV/TV); (d) trabecular thickness (Tb.Th) and (e) trabecular separation (Tb.Sp) of tibia samples from ~6.5‐month‐old *Zmpste24* ^*+/+*^ */RANKL* ^*fl/fl*^ (*n* = 18), *Zmpste24* ^*−/−*^ */RANKL* ^*fl/fl*^ (*n* = 13) and *Zmpste24* ^*−/−*^ */RANKL* ^*fl/fl*^ */Dmp1‐Cre* (*n* = 11) littermates. Graphs show medians and interquartile range. Statistical analysis was performed using the Kruskal‐Wallis test in (b) and (c), one‐way ANOVA test in (d) and (e). *p < 0.05, **p < 0.01, ***p < 0.001, ****p < 0.0001. Outliers identified with the ROUT method (*Q* = 0.1%) were discarded from the analysis.

To determine whether the osteoporotic phenotype in *Zmpste24* ^*−/−*^ progeroid mice could be ameliorated by targeting the cytokine RANKL, we sought to eliminate RANKL production specifically in osteocytes of *Zmpste24* ^*−/−*^ mice. Although several cellular sources of RANKL have been described, previous research has demonstrated that osteocyte‐derived RANKL deletion does not affect bone development but modulates bone remodeling during adulthood (Xiong et al. 2011). Indeed, RANKL deletion in osteocytes has been shown to protect mice from pathological bone loss induced by mechanical unloading (Xiong et al. 2011). To generate osteocyte‐specific RANKL‐deficient progeroid mice, we crossed a mouse strain harboring the *Tnfsf11* allele flanked by *loxP* sites (*RANKL* ^*fl/fl*^) with transgenic mice expressing Cre recombinase under the control of the *Dmp1* promoter, which is active in osteocytes. These mice (*RANKL* ^*fl/fl*^ */Dmp1‐Cre*) were then crossed with progeroid *Zmpste24* ^*−/−*^ animals to generate our experimental mouse model, hereafter referred to as *Zmpste24* ^*−/−*^ */RANKL* ^*fl/fl*^ */Dmp1‐Cre*. To assess whether osteocyte‐derived RANKL deletion affects the bone loss phenotype of the *Zmpste24* ^*−/−*^ progeroid mice, we performed micro‐computed tomography (μCT) analysis on tibias from ~6.5‐month‐old *Zmpste24* ^*+/+*^ */RANKL* ^*fl/fl*^, *Zmpste24* ^*−/−*^ */RANKL* ^*fl/fl*^, and *Zmpste24* ^*−/−*^ */RANKL* ^*fl/fl*^ */Dmp1‐Cre* mice. Morphometric parameters of trabecular and cortical bone regions were analyzed to evaluate microstructural bone organization in all experimental groups. This analysis revealed that *Zmpste24* ^*−/−*^ */RANKL* ^*fl/fl*^ progeroid mice exhibited increased bone loss compared to their wild‐type littermates (*Zmpste24* ^*+/+*^ */RANKL* ^*fl/fl*^), characterized by a significant decrease in trabecular bone mineral density (BMD), bone volume fraction (BV/TV), and trabecular thickness (Tb.Th), along with an increase in trabecular space (Tb.Sp) (Figure 1). Strikingly, osteocyte‐specific deletion of RANKL in *Zmpste24* ^−/−^ mice (*Zmpste24* ^*−/−*^ */RANKL* ^*fl/fl*^ */Dmp1‐Cre* mice) significantly reversed the osteoporotic phenotype compared to *Zmpste24* ^*−/−*^ */RANKL* ^*fl/fl*^ progeroid mice (Figure 1). These results were obtained in both sexes although the effect was slightly more pronounced in female mice (Figure S1). *Zmpste24* ^*−/−*^ */RANKL* ^*fl/fl*^ */Dmp1‐Cre* mice also showed a recovery in morphometric cortical bone parameters, including a significant increase in cortical area (Ct.Ar) compared to *Zmpste24* ^*−/−*^ */RANKL* ^*fl/fl*^ progeroid mice, which leads to a ratio of cortical area to total area (Ct.Ar/Tt.Ar) similar to that of wild‐type mice (Figure S2a–d). Tissue mineral density (TMD) and cortical thickness (Ct.Th) were also significantly increased in *Zmpste24* ^*−/−*^ */RANKL* ^*fl/fl*^ */Dmp1‐Cre* mice compared to *Zmpste24* ^*−/−*^ */RANKL* ^*fl/fl*^ mice (Figure S2e,f). Analysis of vertebral trabecular bone in *Zmpste24* ^*+/+*^ */RANKL* ^*fl/fl*^, *Zmpste24* ^*−/−*^ */RANKL* ^*fl/fl*^, and *Zmpste24* ^*−/−*^ */RANKL* ^*fl/fl*^ */Dmp1‐Cre* mice also revealed a similar effect of osteocyte‐specific RANKL deletion on the spine of *Zmpste24* ^*−/−*^ progeroid mice to that observed in tibial bone (Figure S3a–d). Altogether, these results demonstrate that targeting RANKL is effective in preventing the bone loss phenotype of *Zmpste24* ^−/−^ mice, restoring bone structure in both long bones and vertebrae.

Given the striking recovery of bone mass observed following osteocyte‐derived RANKL deletion in *Zmpste24*‐deficient mice, we sought to analyze whether these changes may have an effect on the levels of circulating markers associated with bone remodeling. No significant differences in serum calcium, phosphorus, or magnesium levels were detected between wild‐type and progeroid mice (Figure S4a–c). Analysis of bone resorption markers C‐terminal telopeptide of type I (CTX‐I) and tartrate‐resistant acid phosphatase 5b (TRAcP 5b) revealed a significant reduction in TRAcP 5b levels in both *Zmpste24* ^*−/−*^ */RANKL* ^*fl/fl*^ and *Zmpste24* ^*−/−*^ */RANKL* ^*fl/fl*^ */Dmp1‐Cre* mice compared with wild‐type controls. This finding is consistent with a previous study reporting a marked reduction in bone cellularity, including osteoclast numbers, in *Zmpste24* ^*−/−*^ mice relative to wild‐type animals (Rivas et al. 2009) (Figure S4d,e). Although circulating levels of bone resorption markers were not significantly different between *Zmpste24* ^*−/−*^ */RANKL* ^*fl/fl*^ and *Zmpste24* ^*−/−*^ */RANKL* ^*fl/fl*^ */Dmp1‐Cre* mice, analysis of the mRNA expression levels of *Rankl* and *Opg* (Osteoprotegerin)—a decoy receptor that binds *Rankl* inhibiting osteoclastogenesis—in tibial samples shows a decreased *Rankl/Opg* ratio in *Zmpste24* ^*−/−*^ */RANKL* ^*fl/fl*^ */Dmp1‐Cre* mice compared to *Zmpste24* ^*−/−*^ */RANKL* ^*fl/fl*^ mice, suggesting a reduction of local bone resorption following osteocyte‐derived RANKL deletion (Figure S5). Interestingly, serum analyses also showed significantly increased circulating levels of the bone remodeling factors bone morphogenetic protein‐2 (BMP‐2) and transforming growth factor‐β1 (TGF‐β1) in *Zmpste24* ^*−/−*^ */RANKL* ^*fl/fl*^ mice compared with wild‐type controls. This increase was partially attenuated by osteocyte‐specific RANKL deletion, although the differences did not reach statistical significance (Figure S4f,g).

### 2.2 Targeting Osteocyte‐Derived RANKL Improves Muscle Function in *Zmpste24* ^*−/−*^ Mice

**FIGURE 2:**
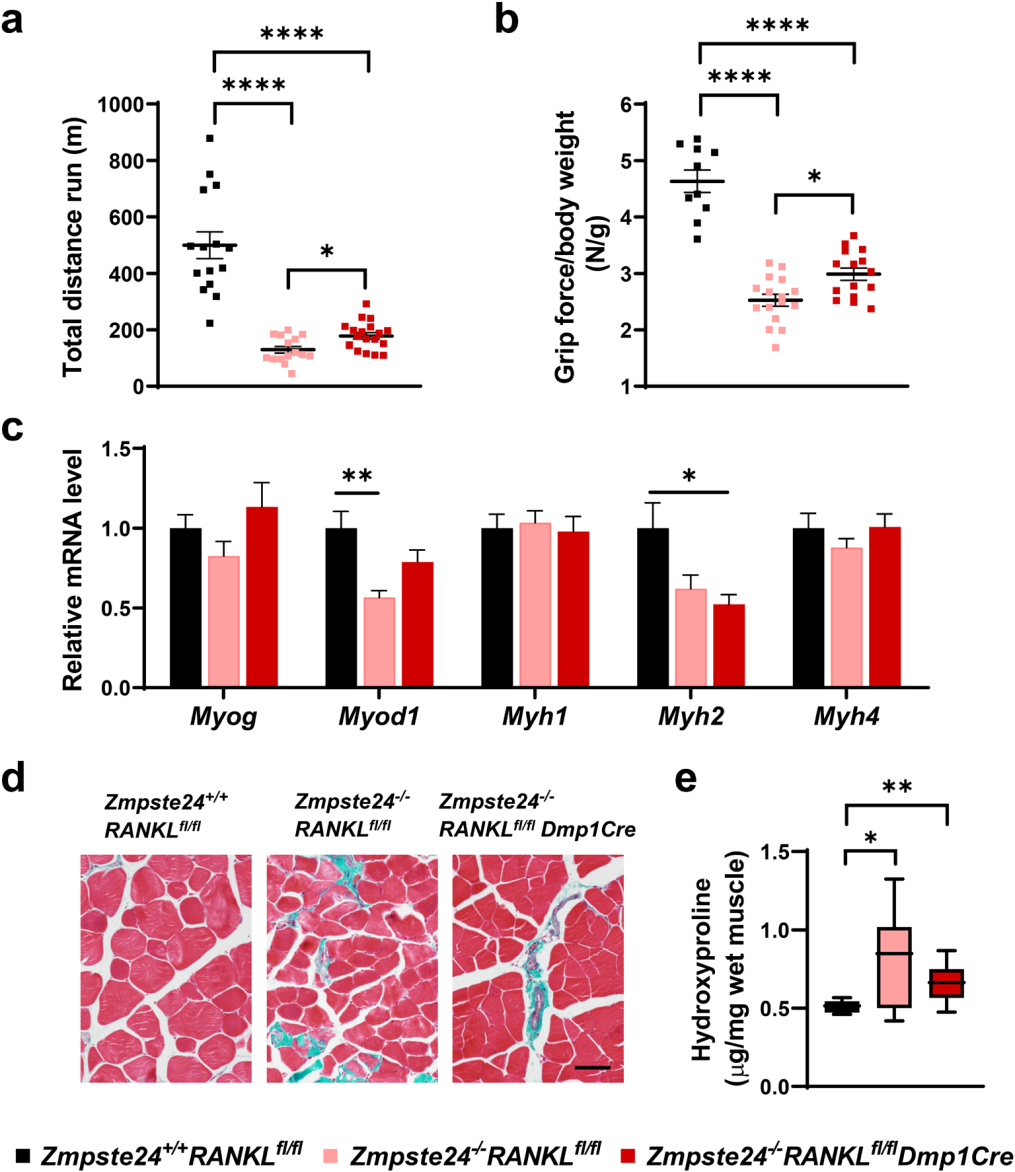
Improved muscle function in *Zmpste24* ^*−/−*^ mice upon osteocyte‐specific RANKL deletion. (a) Total distance run was determined with a treadmill exhaustion test performed in ~5.5‐month‐old *Zmpste24* ^*+/+*^ */RANKL* ^*fl/fl*^ (*n* = 15), *Zmpste24* ^*−/−*^ */RANKL* ^*fl/fl*^ (*n* = 15) and *Zmpste24* ^*−/−*^ */RANKL* ^*fl/fl*^ */Dmp1‐Cre* (*n* = 19) littermates. (b) Forelimb grip force was measured in 5.5‐ to 6‐month‐old *Zmpste24* ^*+/+*^ */RANKL* ^*fl/fl*^ (*n* = 10), *Zmpste24* ^*−/−*^ */RANKL* ^*fl/fl*^ (*n* = 16) and *Zmpste24* ^*−/−*^ */RANKL* ^*fl/fl*^ */Dmp1‐Cre* (*n* = 15) littermates. (c) Relative mRNA expression levels of myogenic and myosin heavy chain (*Myh*) genes in quadriceps samples from ~6‐month‐old *Zmpste24* ^*+/+*^ */RANKL* ^*fl/fl*^ (*n* = 7), *Zmpste24* ^*−/−*^ */RANKL* ^*fl/fl*^ (*n* = 11) and *Zmpste24* ^*−/−*^ */RANKL* ^*fl/fl*^ */Dmp1‐Cre* (*n* = 8) littermates. (d) Fibrosis was assessed by Gomori's trichrome staining in quadriceps samples from ~6.5‐month‐old *Zmpste24* ^*+/+*^ */RANKL* ^*fl/fl*^, *Zmpste24* ^*−/−*^ */RANKL* ^*fl/fl*^ and *Zmpste24* ^*−/−*^ */RANKL* ^*fl/fl*^ */Dmp1‐Cre* littermates. Scale bar: 50 μm. (e) Hydroxyproline content was measured in quadriceps samples from 6.5 to 7‐month‐old *Zmpste24* ^*+/+*^ */RANKL* ^*fl/fl*^ (*n* = 10), *Zmpste24* ^*−/−*^ */RANKL* ^*fl/fl*^ (*n* = 10) and *Zmpste24* ^*−/−*^ */RANKL* ^*fl/fl*^ */Dmp1‐Cre* (*n* = 10) littermates. Graphs show mean ± SEM in (a–c) and boxplots in (e). Statistical analysis was performed using Welch ANOVA test for (a) and (e), and one‐way ANOVA test for (b) and (c). *p < 0.05, **p < 0.01, ****p < 0.0001.

In addition to bone loss, muscle wasting has been reported in patients with HGPS (Greising et al. 2012; Hennekam 2006). Likewise, muscle weakness has been observed in MADB when the disease is caused by a homozygous missense *ZMPSTE24* mutation (Ben Yaou et al. 2011). Although skeletal muscle alterations have not been fully characterized in progeroid laminopathies, the availability of preclinical models has provided novel insights into the musculoskeletal features potentially affected in these disorders. Among the various skeletal muscle alterations reported in *Zmpste24* ^−/−^ mice, muscle weakness—a common characteristic of age‐related sarcopenia—has been described (Greising et al. 2012). Given the increased bone mass observed in *Zmpste24*‐deficient mice upon osteocyte‐specific RANKL deletion, and considering the previously reported beneficial effects of RANKL targeting on dystrophic or sarcopenic muscles (Bonnet et al. 2019; Jayash et al. 2023), we next assessed whether RANKL deletion in osteocytes could also influence the motor performance of progeroid *Zmpste24* ^−/−^ mice. To this end, we evaluated the endurance capacity of *Zmpste24* ^*−/−*^ */RANKL* ^*fl/fl*^ */Dmp1‐Cre* and *Zmpste24* ^*−/−*^ */RANKL* ^*fl/fl*^ progeroid mice by conducting a treadmill exhaustion test. Notably, the total distance run was significantly lower in *Zmpste24* ^*−/−*^ */RANKL* ^*fl/fl*^ mice compared to their wild‐type littermates (*Zmpste24* ^*+/+*^ */RANKL* ^*fl/fl*^ mice) (Figure 2a). However, *Zmpste24* ^*−/−*^ */RANKL* ^*fl/fl*^ */Dmp1‐Cre* mice exhibited an improved endurance capacity compared to *Zmpste24* ^*−/−*^ */RANKL* ^*fl/fl*^ animals (Figure 2a). We further assessed muscle function using a grip strength meter. This assay revealed that the average maximum grip force applied to the bar was markedly reduced in *Zmpste24* ^*−/−*^ */RANKL* ^*fl/fl*^ progeroid mice compared to wild‐type controls (Figure 2b). Remarkably, this parameter was also significantly higher in *Zmpste24* ^*−/−*^ */RANKL* ^*fl/fl*^ */Dmp1‐Cre* mice than in their *Zmpste24* ^*−/−*^ */RANKL* ^*fl/fl*^ counterparts (Figure 2b).

To get further insights into the improved muscle function observed in *Zmpste24* ^−/−^ progeroid mice upon osteocyte‐specific RANKL deletion, we analyzed the mRNA expression levels of myosin heavy chain (Myh)‐encoding genes—widely used markers to classify myofiber subtypes—in quadriceps muscles dissected from ~6‐month‐old mice. No major differences were found in the mRNA levels of *Myh1* (expressed in type IIx fibers) or *Myh4* (type IIb fibers) between *Zmpste24* ^*+/+*^ */RANKL* ^*fl/fl*^ and progeroid mice, regardless of RANKL deletion. However, lower expression of *Myh2* (type‐IIa fibers) was detected in *Zmpste24* ^*−/−*^ */RANKL* ^*fl/fl*^ mice compared to *Zmpste24* ^*+/+*^ */RANKL* ^*fl/fl*^ animals, a finding that was not reversed by osteocyte‐specific RANKL deletion (Figure 2c). Interestingly, we also detected a significant decrease in the mRNA expression of *Myod1*—a master regulator of myogenic differentiation involved in the regeneration of adult skeletal muscle—in *Zmpste24* ^*−/−*^ */RANKL* ^*fl/fl*^ compared to *Zmpste24* ^*+/+*^ */RANKL* ^*fl/fl*^ mice. This reduction was partially restored in *Zmpste24* ^*−/−*^ */RANKL* ^*fl/fl*^ */Dmp1‐Cre* mice (Figure 2c). Additionally, histological analysis of quadriceps samples revealed increased muscle tissue fibrosis in *Zmpste24* ^*−/−*^ */RANKL* ^*fl/fl*^ mice compared to *Zmpste24* ^*+/+*^ */RANKL* ^*fl/fl*^ controls, consistent with the elevated hydroxyproline content observed in progeroid mice (Figure 2d,e). Notably, osteocyte‐specific RANKL deletion was found to partially ameliorate the skeletal muscle fibrotic phenotype of *Zmpste24* ^−/−^ progeroid mice (Figure 2d,e).

### 2.3 Analysis of the Vascular Phenotype and Calcium Deposition in *Zmpste24*‐Deficient Mice Upon RANKL Deletion From Osteocytes

**FIGURE 3:**
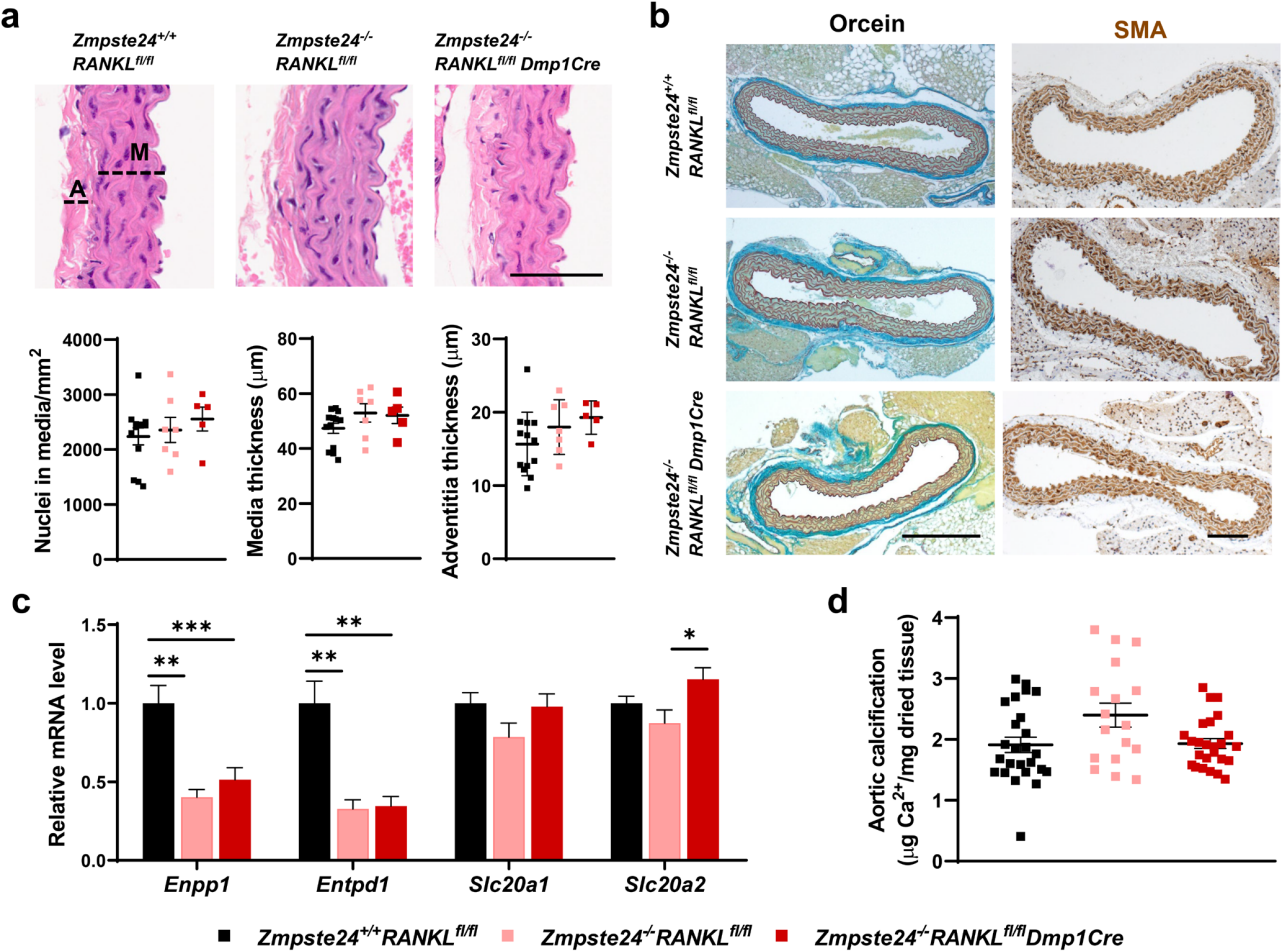
Vascular phenotype and calcium deposition in *Zmpste24*‐deficient mice upon osteocyte‐specific RANKL deletion. (a) Representative aorta sections stained with hematoxylin and eosin. Graphs show quantification of nuclei in the media, media thickness and adventitia thickness of aorta samples from ~6.5‐month‐old *Zmpste24* ^*+/+*^ */RANKL* ^*fl/fl*^ (*n* = 13), *Zmpste24* ^*−/−*^ */RANKL* ^*fl/fl*^ (*n* = 7) and *Zmpste24* ^*−/−*^ */RANKL* ^*fl/fl*^ */Dmp1‐Cre* (*n* = 5) littermates. Scale bar: 50 μm. (B) Representative staining of aorta sections with orcein (left) and anti‐α smooth muscle actin (SMA) antibody (right). Scale bar: 250 μm (left) and 100 μm (right). (c) Relative mRNA expression levels of genes involved in phosphate metabolism in aorta samples from 6.5 to 7‐month‐old *Zmpste24* ^*+/+*^ */RANKL* ^*fl/fl*^ (*n* = 9), *Zmpste24* ^*−/−*^ */RANKL* ^*fl/fl*^ (*n* = 10) and *Zmpste24* ^*−/−*^ */RANKL* ^*fl/fl*^ */Dmp1‐Cre* (*n* = 10) littermates. (d) Calcium content in aorta samples from ~6.5‐month‐old *Zmpste24* ^*+/+*^ */RANKL* ^*fl/fl*^ (*n* = 9), *Zmpste24* ^*−/−*^ */RANKL* ^*fl/fl*^ (*n* = 10) and *Zmpste24* ^*−/−*^ */RANKL* ^*fl/fl*^ */Dmp1‐Cre* (*n* = 10) littermates. Graphs show the means ± SEM. Statistical analysis was performed using Kruskal‐Wallis test and one‐way ANOVA test in (a), Welch ANOVA test and one‐way ANOVA test in (c), and Welch ANOVA test in (d). Outliers identified with the ROUT method (*Q* = 0.1%) were discarded from the analysis in (d). *p < 0.05, **p < 0.01, ***p < 0.001. A indicates adventitia, and M, media.

Cardiovascular disease is the main cause of death in patients with HGPS. Among the pathological features that characterize their vascular disease are the loss of vascular smooth muscle cells (VSMCs), vascular fibrosis, and calcification. Given the pathogenic relationship between altered bone homeostasis and vascular calcification (Persy and D'Haese 2009), we aimed to explore whether improving the osteoporotic phenotype of progeroid mice, following the deletion of RANKL in osteocytes, has any effect on the vasculature of these mice. Histological analysis of aortic sections from ~6.5‐month‐old *Zmpste24* ^*+/+*^ */RANKL* ^*fl/fl*^, *Zmpste24* ^*−/−*^ */RANKL* ^*fl/fl*^ and *Zmpste24* ^*−/−*^ */RANKL* ^*fl/fl*^ */Dmp1‐Cre* mice revealed similar numbers of VSMCs in all experimental groups (Figure 3a), indicating that *Zmpste24* ^*−/−*^ mice, unlike other HGPS mouse models, do not recapitulate this aspect of the vascular disease, in agreement with recently reported data (Kim et al. 2025). We also did not observe significant differences in the thickness of the media or adventitial layers among *Zmpste24* ^*+/+*^ */RANKL* ^*fl/fl*^, *Zmpste24* ^*−/−*^ */RANKL* ^*fl/fl*^ or *Zmpste24* ^*−/−*^ */RANKL* ^*fl/fl*^ */Dmp1‐Cre* mice (Figure 3a). Likewise, the structure of elastic fibers, evaluated with orcein staining, revealed no major histological alterations in progeroid mice, which also preserved α‐smooth muscle actin expression (Figure 3b). However, the analysis of the mRNA levels of genes involved in mineral metabolism revealed significant differences in progeroid *Zmpste24* ^*−/−*^ */RANKL* ^*fl/fl*^ mice compared to *Zmpste24* ^*+/+*^ */RANKL* ^*fl/fl*^ controls (Figure 3c). Thus, we observed markedly lower expression levels of *Enpp1* (ectonucleotide pyrophosphatase/phosphodiesterase 1) in progeroid mice compared to controls. Indeed, loss‐of‐function mutations in this gene in humans and mice cause generalized arterial calcification (Rutsch et al. 2003). However, deletion of osteocyte RANKL did not restore the expression levels of *Enpp1* in *Zmpste24* ^*−/−*^ */RANKL* ^*fl/fl*^ */Dmp1‐Cre* mice. Similar results were found for *Entpd1* (ectonucleoside triphosphate diphosphohydrolase 1) (Figure 3c). No differences were detected in the expression levels of the genes encoding the phosphate transporters *Slc20a1* and *Slc20a2* between *Zmpste24* ^*−/−*^ */RANKL* ^*fl/fl*^ mice and *Zmpste24* ^*+/+*^ */RANKL* ^*fl/fl*^ controls (Figure 3c). Given these findings, we next evaluated vascular calcification by quantifying the calcium content of aortas dissected from ~6.5–month‐old *Zmpste24* ^*+/+*^ */RANKL* ^*fl/fl*^, *Zmpste24* ^*−/−*^ */RANKL* ^*fl/fl*^ and *Zmpste24* ^*−/−*^ */RANKL* ^*fl/fl*^ */Dmp1‐Cre* mice. This analysis showed an increase in the amount of calcium deposited in the aortas of *Zmpste24* ^*−/−*^ */RANKL* ^*fl/fl*^ mice compared to *Zmpste24* ^*+/+*^ */RANKL* ^*fl/fl*^ controls (Figure 3d). Notably, this increase was lowered in *Zmpste24* ^*−/−*^ */RANKL* ^*fl/fl*^ */Dmp1‐Cre* mice, although these differences did not reach statistical significance (Figure 3d).

### 2.4 Osteocyte‐Specific RANKL Deletion Increases Survival of *Zmpste24* ^*−/−*^ Mice

**FIGURE 4:**
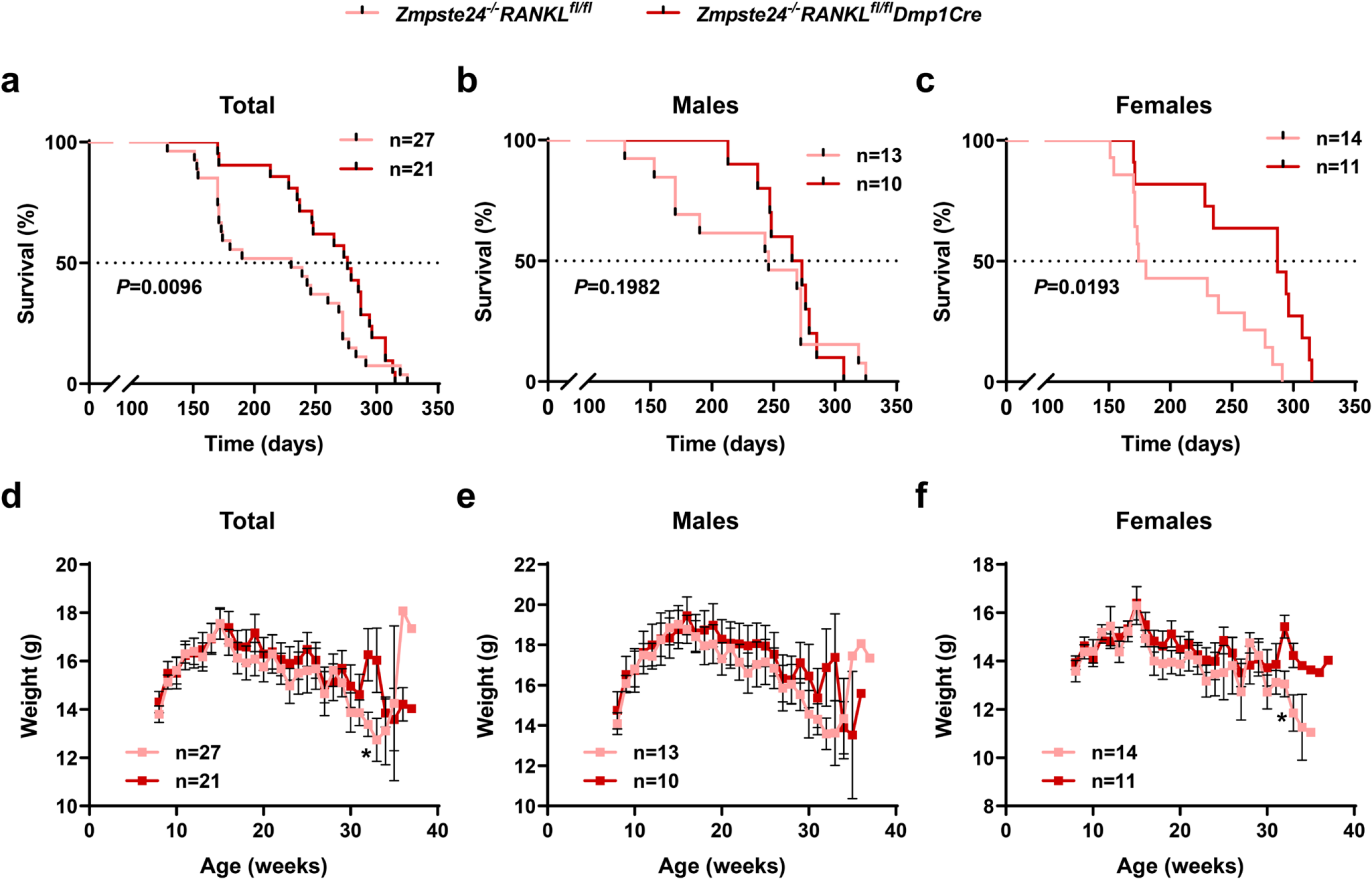
Deletion of osteocyte‐specific RANKL increases the survival of *Zmpste24* ^−/−^ mice. Kaplan–Meier survival curves of total (a), male (b) and female (c) *Zmpste24* ^*−/−*^ */RANKL* ^*fl/fl*^ and *Zmpste24* ^*−/−*^ */RANKL* ^*fl/fl*^ */Dmp1‐Cre* mice. Survival was analyzed with the Gehan‐Breslow‐Wilcoxon test (*p* = 0.0096 for total population, *p* = 0.1982 for males and *p* = 0.0193 for females) and the Log‐rank (Mantel‐Cox) test (*p* = 0.0889 for total population, *p* = 0.6967 for males and *p* = 0.0041 for females). Body weight curves for the total cohort (d), males (e) and females (f) of *Zmpste24* ^*−/−*^ */RANKL* ^*fl/fl*^ and *Zmpste24* ^*−/−*^ */RANKL* ^*fl/fl*^ */Dmp1‐Cre* mice. Statistical analysis was performed using a two‐tailed Student's *t* test. *p < 0.05.

The recovery of bone mass and the improvement in muscle strength, together with the observed trend toward a reduction in calcium deposition in the aortas of *Zmpste24* ^−/−^ mice upon RANKL deletion, prompted us to explore whether our genetic strategy, aimed at targeting osteocyte RANKL and bone resorption, could also have an overall effect on the lifespan of progeroid mice. Notably, analysis of survival curves revealed that *Zmpste24* ^*−/−*^ */RANKL* ^*fl/fl*^ */Dmp1‐Cre* mice showed an increased survival, particularly at early stages, compared to *Zmpste24* ^*−/−*^ */RANKL* ^*fl/fl*^ mice, with a median lifespan of 276 days versus 230 days, respectively, representing a 20% increment (*p* = 0.0096; Gehan‐Breslow‐Wilcoxon test) (Figure 4a). When analyzing the effect on both sexes separately, we observed that the main improvement in survival was achieved in female mice (Figure 4b,c). Thus, although both male and female *Zmpste24* ^*−/−*^ */RANKL* ^*fl/fl*^ */Dmp1‐Cre* mice seemed protected from early death, only female mice showed an increase in their maximum lifespan compared to *Zmpste24* ^*−/−*^ */RANKL* ^*fl/fl*^ mice (Figure 4c). This increased survival at later stages in *Zmpste24* ^*−/−*^ */RANKL* ^*fl/fl*^ */Dmp1‐Cre* female mice was accompanied by a slightly improved body weight, which was not observed in males (Figure 4d–f).

### 2.5 Anti‐RANKL Treatment Improves *Zmpste24* ^*−/−*^ Mice Phenotype and Lifespan

**FIGURE 5:**
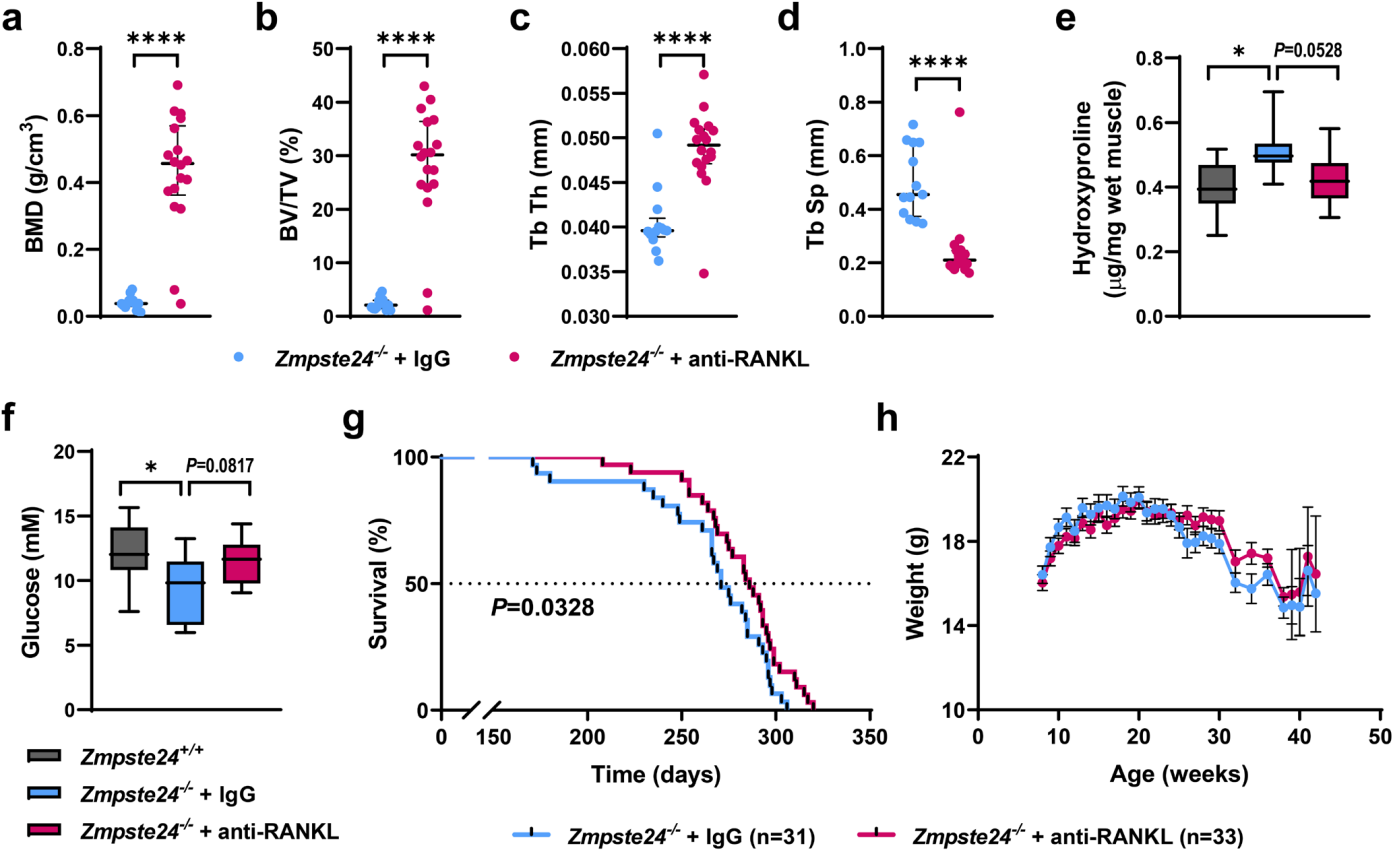
Anti‐RANKL treatment prevents bone loss and extends lifespan in *Zmpste24* ^*−/−*^ mice. (a) Bone mineral density (BMD); (b) bone volume relative to tissue volume (BV/TV); (c) trabecular thickness (Tb.Th) and (d) trabecular separation (Tb.Sp) of tibia samples from *Zmpste24* ^*−/−*^ mice treated with control IgG (*n* = 13) or anti‐RANKL (*n* = 18). (e) Hydroxyproline content was measured in quadriceps samples from ~7.5‐month‐old *Zmpste24* ^*+/+*^ (*n* = 8) and *Zmpste24* ^*−/−*^ mice treated with control IgG (*n* = 11) or anti‐RANKL (*n* = 11). (f) Circulating glucose levels were determined in plasma samples from ~7.5‐month‐old *Zmpste24* ^*+/+*^ (*n* = 8) and *Zmpste24* ^*−/−*^ mice treated with control IgG (*n* = 10) or anti‐RANKL (*n* = 13). Kaplan–Meier survival curve (g) and body weight curve (h) of *Zmpste24* ^*−/−*^ mice treated with control IgG (*n* = 31) or anti‐RANKL (*n* = 33). Survival analysis was performed using the Log‐rank (Mantel‐Cox) test (*p* = 0.0328). Graphs show medians with interquartile range in (a–d) and boxplots in (e, f). Statistical analysis was performed using a two‐tailed Student's *t* test for (a), Mann–Whitney test for (b–d), Kruskal‐Wallis test for (e), and one‐way ANOVA test in (f). *p < 0.05, ****p < 0.0001.

Given the positive effects observed in the progeroid phenotype of *Zmpste24* ^*−/−*^ mice, as a result of the genetic strategy used to delete RANKL from osteocytes, we decided to evaluate whether a standard therapy based on the use of a neutralizing anti‐RANKL antibody could also exert a positive effect on this progeroid mouse model. To this end, *Zmpste24* ^*−/−*^ mice were treated with a monoclonal anti‐mouse RANKL antibody or an isotype control (IgG2a) at a dose of 5 mg/kg twice per week, starting at 8 weeks of age. We first examined the effect of anti‐RANKL therapy on the osteoporotic phenotype of *Zmpste24* ^*−/−*^ progeroid mice. μCT analysis revealed that morphometric trabecular bone parameters—BMD, BV/TV and Tb.Th—were significantly increased in *Zmpste24* ^*−/−*^ mice treated with anti‐RANKL antibody compared to IgG‐treated controls, whereas Tb.Sp was significantly decreased (Figure 5a–d). Likewise, anti‐RANKL treatment also prevented cortical bone loss in *Zmpste24*‐deficient mice (Figure S6). These results indicate that this anti‐osteoporotic therapy, widely used in age‐related conditions, is also effective in the context of progeroid laminopathies. Next, we asked whether anti‐RANKL treatment could have additional benefits beyond those found in bone tissue. We focused on skeletal muscle fibrosis, which had shown the most prominent improvement following osteocyte‐specific RANKL deletion in progeroid mice. Indeed, the hydroxyproline content was notably reduced in the quadriceps muscles of anti‐RANKL‐treated *Zmpste24* ^*−/−*^ mice compared to IgG‐treated controls (Figure 5e). Likewise, given the previously reported role of RANKL in the regulation of glucose metabolism associated to muscle function (Bonnet et al. 2019), we next examined the circulating glucose levels in IgG‐treated and anti‐RANKL‐treated *Zmpste24* ^*−/−*^ mice. Notably, the pathological hypoglycemia characteristic of progeroid *Zmpste24* ^*−/−*^ mice was improved following anti‐RANKL treatment (Figure 5f). Finally, we assessed whether anti‐RANKL treatment could also impact the lifespan of progeroid mice. Remarkably, anti‐RANKL‐treated *Zmpste24* ^*−/−*^ mice showed increased survival compared to IgG‐treated controls (*p* = 0.0328) (Figure 5g). A more pronounced treatment effect was observed in female mice, although the differences between survival curves did not reach statistical significance when both sexes were analyzed separately (Figure S7a,b). No significant differences in body weight were observed between treatment groups when both sexes were analyzed together (Figure 5h), although anti‐RANKL‐treated female *Zmpste24* ^*−/−*^ mice exhibited a slight improvement in body weight compared to IgG‐treated controls when both sexes were analyzed separately (Figure S7c,d).

## 3 Discussion

Osteoporosis is one of the main clinical features of patients with HGPS and other progeroid laminopathies, a characteristic that has been recapitulated in most mouse models generated to study these syndromes (Cubria et al. 2020; Osorio et al. 2011; Varela et al. 2008). In this work, we investigated the effect of targeting the osteokine RANKL, which is involved in bone remodeling, as a potential therapeutic approach to ameliorate the bone loss phenotype in the context of HGPS. Our findings, using both genetic and translational approaches, demonstrate that targeting RANKL effectively improves bone mass in the long bones and vertebrae of progeroid mice. These results align with a substantial body of literature showing the efficacy of different antiresorptive strategies based on RANKL inhibition in naturally occurring, age‐related osteoporosis (Kendler et al. 2022). Similarly, bisphosphonates (such as zoledronic acid) have been shown to improve the bone phenotype in both HGPS mouse models and, more importantly, in patients with HGPS (Cubria et al. 2020; Gordon et al. 2016; Varela et al. 2008). However, these studies were conducted in combination with additional prenylation inhibitors (lonafarnib and pravastatin); as such, the effect of bisphosphonates alone on bone morphometric parameters was not explored. It is important to note that the positive effects may arise not only from their antiresorptive role on bone metabolism but also from their efficacy as prenylation inhibitors. Therefore, to our knowledge, our study provides the first evidence that targeting RANKL, an alternative antiresorptive strategy, yields positive effects in the absence of other treatments. Notably, this approach has been shown to offer additional benefits in different age‐related pathological conditions (Marcadet et al. 2022).

Our results also demonstrate that conditional deletion of osteocyte‐derived RANKL improved the muscle function of progeroid *Zmpste24* ^*−/−*^ mice. We observed significant improvements in endurance exercise capacity and muscle strength in *Zmpste24* ^*−/−*^ mice following osteocyte‐derived RANKL deletion, compared to *Zmpste24* ^*−/−*^ */RANKL* ^*fl/fl*^ mice. Several studies have highlighted the relevance and complexity of bone‐muscle crosstalk beyond mere mechanical loading. Both bone and muscle tissue secrete a wide variety of cytokines (including RANKL), growth factors and extracellular vesicles, which contribute to an intricate network of bidirectional communication essential for their development and homeostasis (Bonewald 2019). Altered crosstalk between bone and muscle has been implicated in the pathophysiology of osteosarcopenia in older people (He et al. 2020). Therefore, our results suggest that the reduced osteoporosis observed in *Zmpste24* ^*−/−*^ mice upon deletion of osteocyte RANKL may have positively impacted neighboring muscle tissues or, alternatively, that RANKL targeting may have directly influenced skeletal muscle homeostasis. Indeed, some studies have highlighted the beneficial effects of RANKL inhibition on muscle function in different pathological conditions. RANKL and its receptor, RANK, are expressed not only in bone but also in skeletal muscle (Bonnet et al. 2019; Dufresne et al. 2018). Both muscle‐specific RANK deletion and RANKL inhibition have been shown to improve muscle performance in mouse models of muscular dystrophy and sarcopenia (Bonnet et al. 2019; Dufresne et al. 2018; Hamoudi et al. 2019; Jayash et al. 2023). Moreover, while bisphosphonates and denosumab are effective drugs approved for the treatment of osteoporosis, denosumab has been shown to exert greater positive effects on muscle function compared to bisphosphonates (Bonnet et al. 2019). Interestingly, several studies have linked the improvement in muscle function upon RANKL inhibition to a reduction in muscle fibrosis (Hamoudi et al. 2019; Jayash et al. 2023). In line with this, we observed a marked decrease in hydroxyproline content in both experimental approaches targeting RANKL in *Zmpste24* ^*−/−*^ mice, suggesting that the reduction in collagen deposition may have contributed to the improved muscle performance observed in progeroid mice upon RANKL inhibition. However, given the complexity of the skeletal muscle phenotype in *Zmpste24* ^*−/−*^ mice and other HGPS mouse models (Greising et al. 2012; Hong et al. 2024), the key crosstalk between bone and muscle, and the growing body of evidence on the role of RANKL in muscle pathology, further studies are needed to evaluate the precise role that this cytokine plays in muscle dysfunction in the context of HGPS.

The relationship between decreased bone mineralization and increased vascular calcification has been established for decades and supported by numerous epidemiological and clinical studies (Persy and D'Haese 2009). However, the mechanisms involved in the development of this vascular condition are complex and diverse (Demer and Tintut 2008). Vascular calcification is one of the clinical features of the cardiovascular disease characteristic of patients with HGPS (Olsen et al. 2023) and has also been reported in patients with MAD (Guo et al. 2025). We found that aortic calcium content was increased in *Zmpste24* ^*−/−*^ */RANKL* ^*fl/fl*^ mice compared to wild‐type controls, consistent with findings in other mouse models of HGPS (Villa‐Bellosta 2019), even though *Zmpste24* ^*−/−*^ mice did not exhibit loss of VSMCs. These results agree with previous studies showing that prelamin A accumulates in calcified arteries, regardless of age, and accelerates mineralization in VSMCs (Liu et al. 2013). Interestingly, we observed a decrease in calcium content in *Zmpste24* ^*−/−*^ mice following osteocyte‐derived RANKL deletion compared to *Zmpste24* ^*−/−*^ */RANKL* ^*fl/fl*^ mice. Among the many factors involved in vascular calcification, the ratio between inorganic phosphate (Pi) and inorganic pyrophosphate (PPi) is critical for the process of mineralization. We found decreased mRNA expression levels of *Enpp1* and *Entpd1*, both enzymes that regulate this ratio and have been implicated in vascular calcification in another mouse model of HGPS (Villa‐Bellosta 2019). However, we did not observe any differences in the expression levels of these enzymes between *Zmpste24* ^*−/−*^ */RANKL* ^*fl/fl*^ and *Zmpste24* ^*−/−*^ */RANKL* ^*fl/fl*^ */Dmp1‐Cre* mice, suggesting that other mechanisms may be involved in the reduction of calcium content observed upon osteocyte RANKL depletion. Indeed, the axis formed by RANK/RANKL and OPG has been directly related to the process of vascular calcification. A study in a knock‐out mouse model for *Opg*, which exhibited both osteoporosis and severe arterial calcification, was the first to suggest a role for this signaling pathway in both bone and vascular diseases (Bucay et al. 1998). Interestingly, the increase in *Rankl/Opg* ratio observed in tibial samples from progeroid *Zmpste24* ^*−/−*^ mice relative to wild‐type controls was primarily driven by a marked reduction in *Opg* mRNA expression. This finding differs from previous observations in osteoblasts from patients with MAD type A (MADA), where *Opg* expression was reported to be increased (Avnet et al. 2011). However, it is important to note that the *Opg* levels measured in our study reflect the combined contribution of multiple bone cell populations rather than osteoblasts alone. Notably, the same study also highlighted a relevant role for TGF‐β signaling in the altered bone turnover associated with MADA. In this context, the significant increase in the serum levels of both TGF‐β1 and BMP‐2 detected in *Zmpste24* ^*−/−*^ */RANKL* ^*fl/fl*^ mice may also be relevant for the development of extra‐skeletal manifestations. Indeed, accumulating evidence indicates that BMP and TGF‐β signaling pathways contribute to pathological bone–vascular crosstalk and vascular remodeling (Iwasaki et al. 2018). BMP‐family members are well‐established drivers of abnormal osteogenic differentiation of vascular cells (Demer and Tintut 2008; Durham et al. 2018). Therefore, the partial normalization of circulating BMP‐2 and TGF‐β1 levels observed in *Zmpste24* ^*−/−*^ */RANKL* ^*fl/fl*^ */Dmp1‐Cre* mice raises the possibility that reducing osteocyte‐derived RANKL not only attenuates local osteoclastogenic signaling but also modulates a broader bone‐derived endocrine environment that may contribute to systemic manifestations of progeria.

Nevertheless, the role of the RANK/RANKL/OPG system in vascular biology has become increasingly complex. Our results align with previous research showing that RANKL inhibition, using denosumab, can reduce vascular calcification in certain pathological contexts (Suzuki et al. 2021). However, other clinical trials targeting RANKL have shown no effect (Samelson et al. 2014), highlighting the importance of the etiology of the vascular disease in treatment outcomes. Further studies are needed to better understand the role of bone metabolism and RANKL in vascular pathology in progeroid laminopathies.

In both humans and mice, various genetic and translational approaches using neutralizing antibodies against RANKL have demonstrated the benefits of targeting this pathway in bone, muscular, and vascular pathologies. Our study shows that this strategy goes beyond improving these conditions in progeria, leading to an increase in the survival of *Zmpste24* ^*−/−*^ mice. Interestingly, a recent study reported that neutralization of IL‐6, a potent inducer of RANKL production, ameliorates several manifestations of premature aging, including lifespan (Squarzoni et al. 2021). Several factors could explain the positive outcome of targeting RANKL in progeria. Increasing evidence suggests that the bone tissue plays important roles beyond its structural functions, acting as both an endocrine and paracrine organ. It has been shown that bone‐derived factors regulate many organs and tissues, including (but not limited to) muscle, liver, kidney, pancreas, brain, and the circulatory and immune systems, highlighting its broad impact on body homeostasis (He et al. 2025). Indeed, we observed an amelioration of hypoglycemia in *Zmpste24* ^*−/−*^ mice upon anti‐RANKL treatment. Therefore, it is conceivable that the recovery of the osteoporotic phenotype in *Zmpste24* ^*−/−*^ mice upon RANKL targeting may have impacted the secretome of progeroid mice, locally or systemically, which has been previously shown to be profoundly altered compared to wild‐type mice (Quintana‐Torres et al. 2023). Similarly, the beneficial effects observed on muscle fibrosis—potentially derived from the recovery of bone mass and its positive crosstalk with neighboring muscle tissue, or through a direct action of RANKL signaling on muscle biology—also raise the possibility that improvements in physical activity may have further enhanced the overall health of progeroid mice. The systemic benefits of physical exercise on healthspan have been widely reported in both humans and mice (Zhao et al. 2020). Furthermore, we cannot rule out the impact of the reduction in vascular calcium deposition observed in *Zmpste24* ^*−/−*^ */RANKL* ^*fl/fl*^ */Dmp1‐Cre* mice on the survival of these progeroid mice. A previous study in another HGPS preclinical model demonstrated that a combined treatment aimed at preventing vascular calcification extended its lifespan, underscoring the importance of vascular calcification in HGPS (Villa‐Bellosta 2019). Nevertheless, although downstream pathways can be therapeutically targeted, the most pronounced lifespan extensions reported to date in progeroid mouse models have been achieved by interventions that correct or counteract the underlying pathogenic mutation. Therefore, given the multifactorial nature of the disease, targeting a single downstream pathway is unlikely to correct the pathological phenotype.

In summary, we have demonstrated that RANKL targeting is an effective strategy to rescue the osteoporotic phenotype of progeroid *Zmpste24* ^*−/−*^ mice, as well as to provide additional benefits on extra‐osseous tissues, ultimately improving survival. Further in‐depth studies are needed to assess the full potential of RANKL inhibition as a therapeutic approach for treating HGPS.

## 4 Experimental Procedures

### 4.1 Animal Experiments

All animal procedures were performed in accordance with the guidelines of the Committee for Animal Experimentation at the Universidad de Oviedo (Oviedo, Spain) and were approved by the *Consejería de Desarrollo Rural y Recursos Naturales del Principado de Asturias* (PROAE 14/2018, PROAE 41/2019 and PROAE 27/2024). *Zmpste24* ^*−/−*^ mouse model, previously generated in our laboratory (Pendas et al. 2002), was maintained on a mixed C57BL/6NCrl‐C57BL/6J background. *RANKL* ^*fl/fl*^ (Xiong et al. 2011) and *Dmp1‐Cre* (Lu et al. 2007) mice were obtained from Jackson Laboratories (Strains: #018978 and #023047, respectively). Mice carrying the *Cre* allele were always maintained in hemizygosity. Animals were housed in a pathogen‐free facility under controlled environmental conditions: a 12 h light/12 h dark cycle, a temperature of 22°C ± 2°C, and relative humidity of 50% ± 10%. Mice were monitored daily for good physical condition. To support feeding in progeroid mice, softened food pellets were provided. For the experiments involving RANKL neutralization, Z*mpste24* ^*−/−*^ mice were subcutaneously injected twice per week, starting at 8 weeks of age, with either the InVivoMAb anti‐mouse RANKL monoclonal antibody (clone IK22/5, BE0191, BioXCell) or rat IgG2a control type (BE0089, BioXCell), at a dose of 5 mg/kg. Antibodies were dissolved in PBS.

### 4.2 Micro‐Computed Tomography Analysis

To analyze trabecular architecture, tibiae and column were dissected from ~6.5‐month‐old *Zmpste24* ^*−/−*^ */RANKL* ^*fl/fl*^ */Dmp1‐Cre* mice and their corresponding littermate controls, as well as tibiae from endpoint Z*mpste24* ^*−/−*^ mice treated with anti‐RANKL or IgG2a control antibody. Samples were fixed in 4% buffered formaldehyde solution and stored at 4°C in 90% ethanol until analysis. The region extending from the proximal metaphysis to the mid‐diaphysis of tibia specimens and L4 vertebrae was scanned using high‐resolution micro‐computed tomography (μCT) (SkyScan 1174, SkyScan, Bruker, Kontich, Belgium). Scans were acquired using a 50 kV X ray tube voltage, 800 μA current, and a 0.5 mm aluminum filter. Image reconstruction was performed using NRecon software (SkyScan). For morphometric analysis in 2D and 3D, we used the software provided by the manufacturer (CTAn). The region of interest (ROI) was manually defined for each sample. For the analysis of the diaphyseal cortical region of tibiae, 100 slices were selected. Global grayscale threshold levels for this area were set between 95 and 255. For the trabecular region, a total of 150 slices were selected and adaptative grayscale threshold levels between 63 and 255 were used. The morphometric parameters determined were bone mineral density (BMD), ratio of bone volume/tissue volume (BV/TV), trabecular thickness (Tb.Th), and trabecular separation (Tb.Sp) for the trabecular area. For the cortical region, tissue mineral density (TMD), cortical thickness (Ct.Th), total area (Tt.Ar), cortical area (Ct.Ar), medullary area (Ma.Ar), and the ratio of cortical area to total area (Ct.Ar/Tt.Ar) were examined.

### 4.3 Treadmill Exhaustion Test

Endurance capacity was evaluated using an incremental treadmill protocol (Panlab 8700, Barcelona, Spain) set at a constant 10° slope. Mice began running at 0.10 m/s, with the speed increased by 0.025 m/s every 3 min until exhaustion. Total distance run (meters) was determined to evaluate endurance performance. Prior to testing, mice underwent a 5‐day progressive adaptation period to treadmill exercise, during which they were briefly exposed to low‐intensity running to acclimate them to the treadmill environment.

### 4.4 Grip Strength Test

Forelimb grip force was assessed using a digital grip strength meter (Harvard Apparatus). Mice were gently held by the tail and allowed to grasp a horizontal bar with their forepaws. The grip force exerted when mice were pulled backwards was measured. This procedure was repeated three times, and the maximum grip force out of the three attempts was recorded. The test was performed on three consecutive days. For each mouse, the mean maximum grip force was calculated and normalized to mouse body mass.

### 4.5 Histological Analysis

Tissues were fixed in 4% buffered formaldehyde solution overnight at 4°C and embedded in paraffin by standard procedures. Thoracic aorta tissue sections were stained with hematoxylin and eosin (H&E) to quantify the number of vascular smooth muscle cells (VSMCs). Orcein staining was used to evaluate elastic fibers. Aortic media and adventitia thickness were measured in 3 different fields per sample and mean values were calculated for each mouse. Immunohistochemistry staining was performed in mouse aortas using a rabbit polyclonal anti‐alpha smooth muscle actin (SMA) antibody (ab5694, Abcam). Gomori's trichrome staining was used to assess collagen deposition in quadriceps muscle.

### 4.6 Quantification of Aortic Calcium Content

Aortic calcium content was quantified as previously described (Villa‐Bellosta 2019). Briefly, thoracic aortas were dissected from ~6.5‐month‐old *Zmpste24* ^*−/−*^ */RANKL* ^*fl/fl*^ */Dmp1‐Cre* mice and their corresponding littermate controls, minced, and dried overnight at 55°C. After dehydration, aortic samples were weighed and treated with 0.5 mol/L HCl for three days at 4°C. Samples were vortexed and spun down once per day. After three days, the resulting supernatant was recovered and used to determine the aortic calcium content using the colorimetric QuantiChrom calcium assay kit (BioAssay Systems).

### 4.7 Hydroxyproline Assay

Hydroxyproline content was biochemically measured in quadriceps muscles dissected from 6.5 to 7‐month‐old *Zmpste24* ^*−/−*^ */RANKL* ^*fl/fl*^ */Dmp1‐Cre* mice and their controls, and 8‐month‐old Z*mpste24* ^*−/−*^ mice treated with anti‐RANKL or IgG2a antibody. Briefly, 50 mg of minced quadriceps samples were homogenized in 500 μL of purified water. 50 μL of 10 N NaOH was added to 50 μL of each tissue homogenate and incubated at 120°C for 1 h. Hydrolyzed samples were cooled to room temperature and neutralized with 50 μL of 10N HCl. Samples were diluted 1:1 with 150 μL of purified water and centrifuged at 14,000× *g* for 2 min to pellet debris. Hydroxyproline content in hydrolyzed samples was determined using the colorimetric Hydroxyproline Assay Kit (Sigma‐Aldrich).

### 4.8 Blood Analyses

Blood was extracted directly from the heart after anesthetizing the mice. Blood was allowed to clot for 3–4 h and centrifuged at 1000× *g* at 4°C for 15 min to obtain serum samples. Harvested serum was stored at −80°C until analysis. Serum levels of calcium, phosphorus, and magnesium were determined by INDILAB laboratories (León, Spain). Commercial enzyme‐linked immunosorbent assay kits were used to evaluate the levels of the following proteins in serum samples: BMP‐2 (DBP200, R&D Systems), TGF‐β1 (DB100C, R&D Systems), and TRAcP 5b (SBTR103, Immunodiagnostic Systems). CTX‐I determination was performed using the Elabscience Mouse CTX I ELISA kit (E‐EL‐M3023) in plasma‐EDTA samples obtained following blood centrifugation at 2200× *g* at 4°C for 15 min. Glucose levels were determined by INDILAB laboratories (León, Spain) using heparinized plasma samples obtained following blood centrifugation at 1000× *g* at 4°C.

### 4.9 RNA Isolation for Real‐Time Quantitative PCR

Quadriceps muscle and thoracic aorta tissues were dissected, snap‐frozen and stored at −80°C. Tibiae were dissected, and the distal and proximal ends were removed prior to centrifugation at 10,000 rpm for 15 s to flush out bone marrow cells. Following centrifugation, the tibiae were snap‐frozen and stored at −80°C. Total RNA was extracted with TRIzol (Life Technologies) or QiAzol (Qiagen) reagents. cDNA was synthesized from total RNA using the QuantiTect Reverse Transcription Kit (Qiagen) or NZY RT SuperMix 5x (NZYtech), according to the manufacturer's instructions. Real‐time quantitative PCR was performed using Power SYBR Green PCR Master Mix (Life Technologies) on a QuantStudio 5 Real‐Time PCR system (Applied Biosystems). All reactions were carried out in triplicate. Relative mRNA levels were calculated using the expression of ribosomal protein lateral stalk subunit P0 (*Rplp0*) as the endogenous reference gene. Primer sequences are listed in Table S1.

### 4.10 Statistical Analysis

Results are presented either as individual data points, bar graphs showing mean ± standard error of the mean (SEM), or boxplots indicating the interquartile range, as specified in the figure legends. Data were assessed for normality using the Shapiro–Wilk test. Two‐group comparisons were performed using the parametric two‐tailed Student's *t*‐test, with Welch's correction if the groups had unequal variances, or the non‐parametric Mann–Whitney test. For multiple group comparison, the parametric one‐way ANOVA test or its non‐parametric equivalent (Kruskal‐Wallis) test was used. Welch ANOVA test was used for variables with different variances. Gehan‐Breslow‐Wilcoxon test and Log‐rank (Mantel‐Cox) test were used for survival analysis. The level of significance was set at 0.05. Statistically significant differences between mouse groups are shown with asterisks (*p < 0.05, **p < 0.01, ***p < 0.001, ***p < 0.0001). The ROUT method (*Q* = 0.1%) was used to detect and clear outliers in those data sets indicated in the figure legends. Statistical tests were performed using GraphPad Prism 8.0 software.

## Author Contributions

Contribution: S.F.‐R., A.R.F., B.F.‐G., C.L.‐O. and A.L.‐S. designed the experiments. S.F.‐R., A.V.‐C., F.R., M.F.‐separationS., B.F.‐G. and V.L. performed the experiments and analyzed the data. M.T.F.‐G. performed histopathological analysis. A.R.F. and A.L.‐S. wrote the manuscript. A.L. and C.F.‐L. contributed to data interpretation and writing of the manuscript.

## Funding

This work was supported by Ministerio de Ciencia e Innovación (RTI2018‐096479‐A‐I00 and PID2021‐126372OB‐I00), Agencia de Ciencia, Competitividad Empresarial e Innovación Asturiana‐SEKUENS (GRU‐GIC‐24‐103), Consejería de Ciencia, Innovación y Universidad del Gobierno del Principado de Asturias (AYUD/2021/51062), and European Research Council (742067, DeAge, ERC‐2016‐ADG). A.V.‐C. is supported by an FPI fellowship from Ministerio de Ciencia e Innovación (RTI2018‐096479‐A‐I00). The IUOPA is funded by the Asturian Government and Fundación Cajastur‐Liberbank.

## Conflicts of Interest

The authors declare no conflicts of interest.

## Supporting information


**Table S1:** Primer sequences used for real‐time quantitative PCR.

**Figure S1:** Deletion of osteocyte RANKL in *Zmpste24*‐deficient mice prevents bone loss in both male and female progeroid mice. (a) Bone mineral density (BMD); (b) bone volume relative to tissue volume (BV/TV); (c) trabecular thickness (Tb.Th) and (d) trabecular separation (Tb.Sp) of tibia samples from ~6.5‐month‐old male *Zmpste24* ^*+/+*^ */RANKL* ^*fl/fl*^ (*n* = 10), *Zmpste24* ^*−/−*^ */RANKL* ^*fl/fl*^ (*n* = 4) and *Zmpste24* ^*−/−*^ */RANKL* ^*fl/fl*^ */Dmp1‐Cre* (*n* = 5) littermates (left panels) and female *Zmpste24* ^*+/+*^ */RANKL* ^*fl/fl*^ (*n* = 8), *Zmpste24* ^*−/−*^ */RANKL* ^*fl/fl*^ (*n* = 9) and *Zmpste24* ^*−/−*^ */RANKL* ^*fl/fl*^ */Dmp1‐Cre* (*n* = 6) littermates (right panels). Graphs show medians and interquartile ranges. Statistical analysis was performed using the Kruskal‐Wallis test and the Welch ANOVA test in (a) and (b), the Kruskal‐Wallis test and one‐way ANOVA test in (c), and the Kruskal‐Wallis test in (d). *p < 0.05, **p < 0.01, ***p < 0.001, ****p < 0.0001.
**Figure S2:** Genetic ablation of osteocyte RANKL protects against cortical bone loss in *Zmpste24*‐deficient mice. (a) Total area (Tt.Ar); (b) cortical area (Ct.Ar); (c) medullary area (Ma.Ar); (d) ratio of cortical area to total area (Ct.Ar/Tt.Ar); (e) tissue mineral density (TMD) and (f) cortical thickness (Ct.Th) of tibia samples from ~6.5‐month‐old *Zmpste24* ^*+/+*^ */RANKL* ^*fl/fl*^ (*n* = 13), *Zmpste24* ^*−/−*^ */RANKL* ^*fl/fl*^ (*n* = 12) and *Zmpste24* ^*−/−*^ */RANKL* ^*fl/fl*^ */Dmp1‐Cre* (*n* = 10) littermates. Graphs show the means ± SEM. Statistical analysis was performed using a one‐way ANOVA test. *p < 0.05, **p < 0.01, ***p < 0.001, ****p < 0.0001.
**Figure S3:** Deletion of osteocyte RANKL in *Zmpste24*‐deficient mice improves bone trabecular structure in the vertebrae. (a) Bone mineral density (BMD); (b) bone volume relative to tissue volume (BV/TV); (c) trabecular thickness (Tb.Th) and (d) trabecular separation (Tb.Sp) of L4 vertebrae from ~6.5‐month‐old *Zmpste24* ^*+/+*^ */RANKL* ^*fl/fl*^ (*n* = 8), *Zmpste24* ^*−/−*^ */RANKL* ^*fl/fl*^ (*n* = 6) and *Zmpste24* ^*−/−*^ */RANKL* ^*fl/fl*^ */Dmp1‐Cre* (*n* = 6) littermates. Graphs show the means ± SEM. Statistical analysis was performed using a one‐way ANOVA test in (a), (b) and (d) and Welch ANOVA test in (c). *p < 0.05, **p < 0.01, ***p < 0.001, ****p < 0.0001.
**Figure S4:** Effects of osteocyte‐derived RANKL deletion on circulating markers of bone remodeling in *Zmpste24*‐deficient mice. Circulating levels of (a) calcium, (b) phosphorus, (c) magnesium, (d) tartrate‐resistant acid phosphatase 5b (TRAcP 5b), (e) C‐terminal telopeptide of type I collagen (CTX‐I), (f) bone morphogenetic protein‐2 (BMP‐2) and (g) transforming growth factor‐β1 (TGF‐β1) were determined in ~6.5‐month‐old *Zmpste24* ^*+/+*^ */RANKL* ^*fl/fl*^ (*n* = 12–17), *Zmpste24* ^*−/−*^ */RANKL* ^*fl/fl*^ (*n* = 7–11) and *Zmpste24* ^*−/−*^ */RANKL* ^*fl/fl*^ */Dmp1‐Cre* (*n* = 6–13) littermates. Graphs show the means ± SEM. Statistical analysis was performed using a one‐way ANOVA test in (a), (c) and (g), and the Welch ANOVA test in (b), (d), (e) and (f). *p < 0.05, **p < 0.01, ***p < 0.001, ****p < 0.0001.
**Figure S5:** Deletion of osteocyte RANKL reduces the *Rankl/Opg* ratio in bone from *Zmpste24*‐deficient mice. Relative mRNA levels of (a) *Rankl* and (b) *Opg*, and (c) *Rankl/Opg* ratio in tibial samples from ~6.5‐month‐old *Zmpste24* ^*+/+*^ */RANKL* ^*fl/fl*^ (*n* = 8–10), *Zmpste24* ^*−/−*^ */RANKL* ^*fl/fl*^ (*n* = 5–6) and *Zmpste24* ^*−/−*^ */RANKL* ^*fl/fl*^ */Dmp1‐Cre* (*n* = 6–8) littermates. Graphs show the means ± SEM. One outlier identified with the ROUT method (*Q* = 0.1%) was discarded from the analysis in (b). Statistical analysis was performed using a one‐way ANOVA test in (a) and the Kruskal‐Wallis test in (b, c). *p < 0.05.
**Figure S6:** Anti‐RANKL treatment prevents cortical bone loss in *Zmpste24*‐deficient mice. (a) Total area (Tt.Ar); (b) cortical area (Ct.Ar); (c) medullary area (Ma.Ar); (d) ratio of cortical area to total area (Ct.Ar/Tt.Ar); (e) tissue mineral density (TMD) and (f) cortical thickness (Ct.Th) of tibia samples from *Zmpste24* ^*−/−*^ mice treated with control IgG (*n* = 12) or anti‐RANKL (*n* = 18). Statistical analysis was performed using a two‐tailed Student's *t* test for (a–c) and Mann–Whitney test for (d–f). *p < 0.05, ****p < 0.0001.
**Figure S7:** Effect of anti‐RANKL treatment on the lifespan of male and female *Zmpste24* ^*−/−*^ mice. Kaplan–Meier survival curve (a) and body weight curve (c) of male *Zmpste24* ^*−/−*^ mice treated with control IgG (*n* = 18) or anti‐RANKL antibody (*n* = 18). Kaplan–Meier survival curve (b) and body weight curve (d) of female *Zmpste24* ^*−/−*^ mice treated with control IgG (*n* = 13) or anti‐RANKL antibody (*n* = 15). Survival curves were analyzed using the Log‐rank (Mantel‐Cox) test. Graphs show the means ± SEM. Statistical analysis was performed using a two‐tailed Student's *t* test in (c) and (d). *p < 0.05.

## Data Availability

The data that support the findings of this study are available from the corresponding author upon reasonable request.
