# Supplementary material for: Targeting RANKL Prevents Bone Loss, Improves Muscle Function and Extends Lifespan in Progeroid Mice
Source: Aging Cell. 2026 Aug 9;25(8):e70655. doi: 10.1111/acel.70655 (PMC13454561; doi:10.1111/acel.70655)
Supplement: Supplementary file 1 [file ACEL-25-e70655-s001.docx]

**c**

**Males**

**Females**

**b**

**Males**

**Females**

**d**

**Males**

**Females**

**a**

**Males**

**Females**

**Supplementary Figure 1. Deletion of osteocyte RANKL in *Zmpste24*-deficient mice prevents bone loss in both male and female progeroid mice.** (a) Bone mineral density (BMD); (b) bone volume relative to tissue volume (BV/TV); (c) trabecular thickness (Tb.Th) and (d) trabecular separation (Tb.Sp) of tibia samples from ~6.5-month-old male *Zmpste24^+/+^/RANKL^fl/fl^* (n=10), *Zmpste24^-/-^/RANKL^fl/fl^* (n=4) and *Zmpste24^-/-^/RANKL^fl/fl^/Dmp1-Cre* (n=5) littermates (left panels) and female *Zmpste24^+/+^/RANKL^fl/fl^* (n=8), *Zmpste24^-/-^/RANKL^fl/fl^* (n=9) and *Zmpste24^-/-^/RANKL^fl/fl^/Dmp1-Cre* (n=6) littermates (right panels). Graphs show medians and interquartile ranges. Statistical analysis was performed using the Kruskal-Wallis test and the Welch ANOVA test in (a) and (b), the Kruskal-Wallis test and one-way ANOVA test in (c), and the Kruskal-Wallis test in (d). **P*˂0.05, ***P*˂0.01, ****P*˂0.001, *****P*˂0.0001.

**Figure S1**

**Figure S2**

**a**

**c**

**e**

**f**

**b**

**d**

**Supplementary Figure 2. Genetic ablation of osteocyte RANKL protects against cortical bone loss in *Zmpste24*-deficient mice.** (a) Total area (Tt.Ar); (b) cortical area (Ct.Ar); (c) medullary area (Ma.Ar); (d) ratio of cortical area to total area (Ct.Ar/Tt.Ar); (e) tissue mineral density (TMD) and (f) cortical thickness (Ct.Th) of tibia samples from ~6.5-month-old *Zmpste24^+/+^/RANKL^fl/fl^* (n=13), *Zmpste24^-/-^/RANKL^fl/fl^* (n=12) and *Zmpste24^-/-^/RANKL^fl/fl^/Dmp1-Cre* (n=10) littermates. Graphs show the means ± SEM. Statistical analysis was performed using a one-way ANOVA test. **P*˂0.05, ***P*˂0.01, ****P*˂0.001, *****P*˂0.0001.

**Figure S3**

**a**

**b**

**c**

**d**

**Supplementary Figure 3. Deletion of osteocyte RANKL in *Zmpste24*-deficient mice improves bone trabecular structure in the vertebrae.** (a) Bone mineral density (BMD); (b) bone volume relative to tissue volume (BV/TV); (c) trabecular thickness (Tb.Th) and (d) trabecular separation (Tb.Sp) of L4 vertebrae from ~6.5-month-old *Zmpste24^+/+^/RANKL^fl/fl^* (n=8), *Zmpste24^-/-^/RANKL^fl/fl^* (n=6) and *Zmpste24^-/-^/RANKL^fl/fl^/Dmp1-Cre* (n=6) littermates. Graphs show the means ± SEM. Statistical analysis was performed using a one-way ANOVA test in (a), (b) and (d) and Welch ANOVA test in (c). **P*˂0.05, ***P*˂0.01, ****P*˂0.001, *****P*˂0.0001.

**Figure S4**

**a**

**b**

**c**

**d**

**e**

**f**

**g**

**Supplementary Figure S4. Effects of osteocyte-derived RANKL deletion on circulating markers of bone remodeling in *Zmpste24*-deficient mice.** Circulating levels of (a) calcium, (b) phosphorus, (c) magnesium, (d) tartrate-resistant acid phosphatase 5b (TRAcP 5b), (e) C-terminal telopeptide of type I collagen (CTX-I), (f) bone morphogenetic protein-2 (BMP-2) and (g) transforming growth factor-β1 (TGF-β1) were determined in ~6.5-month-old *Zmpste24^+/+^/RANKL^fl/fl^* (n=12-17), *Zmpste24^-/-^/RANKL^fl/fl^* (n=7-11) and *Zmpste24^-/-^/RANKL^fl/fl^/Dmp1-Cre* (n=6-13) littermates. Graphs show the means ± SEM. Statistical analysis was performed using a one-way ANOVA test in (a), (c) and (g), and the Welch ANOVA test in (b), (d), (e) and (f). **P*˂0.05, ***P*˂0.01, ****P*˂0.001, *****P*˂0.0001.

**Figure S5**

**a**

**b**

**c**

**Supplementary Figure S5. Deletion of osteocyte RANKL reduces the *Rankl/Opg* ratio in bone from *Zmpste24*-deficient mice.** Relative mRNA levels of (a) *Rankl* and (b) *Opg*, and (c) *Rankl/Opg* ratio in tibial samples from ~6.5-month-old *Zmpste24^+/+^/RANKL^fl/fl^* (n=8-10), *Zmpste24^-/-^/RANKL^fl/fl^* (n=5-6) and *Zmpste24^-/-^/RANKL^fl/fl^/Dmp1-Cre* (n=6-8) littermates. Graphs show the means ± SEM. One outlier identified with the ROUT method (Q=0.1%) was discarded from the analysis in (b). Statistical analysis was performed using a one-way ANOVA test in (a) and the Kruskal-Wallis test in (b,c). **P*˂0.05.

**a**

**c**

**b**

**Figure S6**

**e**

**f**

**d**

**Supplementary Figure S6. Anti-RANKL treatment prevents cortical bone loss in *Zmpste24*-deficient mice.** (a) Total area (Tt.Ar); (b) cortical area (Ct.Ar); (c) medullary area (Ma.Ar); (d) ratio of cortical area to total area (Ct.Ar/Tt.Ar); (e) tissue mineral density (TMD) and (f) cortical thickness (Ct.Th) of tibia samples from *Zmpste24^-/-^* mice treated with control IgG (n=12) or anti-RANKL (n=18). Statistical analysis was performed using a two-tailed Student’s t test for (a-c) and Mann-Whitney test for (d-f). *P˂0.05, ****P˂0.0001.

**Figure S7**

**a**

**b**

**Males**

**Females**

***P=*0.1537**

***P=*0.2420**

**c**

**d**

**Males**

**Females**

*****

*****

*****

**Supplementary Figure 7. Effect of anti-RANKL treatment on the lifespan of male and female *Zmpste24^-/-^* mice.** Kaplan-Meier survival curve (a) and body weight curve (c) of male *Zmpste24^-/-^* mice treated with control IgG (n=18) or anti-RANKL antibody (n=18). Kaplan-Meier survival curve (b) and body weight curve (d) of female *Zmpste24^-/-^* mice treated with control IgG (n=13) or anti-RANKL antibody (n=15). Survival curves were analyzed using the Log-rank (Mantel-Cox) test. Graphs show the means ± SEM. Statistical analysis was performed using a two-tailed Student’s *t* test in (c) and (d). **P*˂0.05.
