# Supplementary material for: Targeting RANKL Prevents Bone Loss, Improves Muscle Function and Extends Lifespan in Progeroid Mice
Source: Aging Cell. 2026 Aug 9;25(8):e70655. doi: 10.1111/acel.70655 (PMC13454561; doi:10.1111/acel.70655)
Supplement: Supplementary file 2 [file ACEL-25-e70655-s002.docx]

**Supplementary Table 1. Primer sequences used for real-time quantitative PCR**

| **Gene** | **Forward primer (5’-3’)** | **Reverse primer (5’-3’)** |
| --- | --- | --- |
| ***Myog*** | CATCCAGTACATTGAGCGCCTA | GAGCAAATGATCTCCTGGGTTG |
| ***Myod1*** | TACAGTGGCGACTCAGATGC | GAGATGCGCTCCACTATGCT |
| ***Myh1*** | GTTCCTCCTTCCAGACCGTG | GGGGATGATGCACCGTACAA |
| ***Myh2*** | ACTTTGGCACTACGGGGAAAC | CAGCAGCATTTCGATCAGCTC |
| ***Myh4*** | TTGAAAAGACGAAGCAGCGAC | AGAGAGCGGGACTCCTTCTG |
| ***Enpp1*** | TGGATTGTGCCAATCAAGG | CTGTTGCTGCTGGAGTAGAC |
| ***Entpd1*** | AGCCTCCACACAGATCACCTT | GCCACCACTTGAAACCTGAAT |
| ***Opg*** | TGCTGAAGCTGTGGAAACATC | AGAAGCTGCTCTGTGGTGAG |
| ***Slc20a1*** | ATTGTCGGTGCAACCATTG | TAATACCAGAAAGCAGCGGA |
| ***Slc20a2*** | CATCGTCGGCTCCACCAT | CTGGCGCTCCTGTGTACA |
| ***Rankl*** | CACACTACCTGACTCCTGCA | CACATCCAACCATGAGCCTTC |
| ***Rplp0*** | GCGACCTGGAAGTCCAACTAC | ATCTGCTGCATCTGCTTGGA |
